# Anisotropic Graphene Aerogels with Integrated Metal–Polyphenol Networks and Thermoresponsive Functionality for Recyclable Photocatalytic Wastewater Treatment

**DOI:** 10.3390/nano16070415

**Published:** 2026-03-30

**Authors:** Na Zhang, Guifeng Tang, Nan Xiang, Huajun Sun, Yanan Hu, Chuanxing Wang

**Affiliations:** College of Chemical Engineering, Qingdao University of Science and Technology, Qingdao 266042, China; 13792311519@163.com (N.Z.); 17861627821@163.com (G.T.); xiangnsun@163.com (N.X.); sunhuajun123@163.com (H.S.); 15552178522@163.com (Y.H.)

**Keywords:** anisotropic graphene aerogel, metal–polyphenol network, thermoresponsive adsorption–desorption, photocatalytic degradation, wastewater treatment

## Abstract

Current strategies for treating organic dye wastewater are shifting from single-function removal processes and catalytic degradation methods toward more integrated treatment approaches. This study proposes a multifunctional composite integrating adsorption–photodegradation–intelligent recovery for photodegradation and recovery of methylene blue-contaminated wastewater. By optimizing the preparation process to precisely control the pore size and arrangement of the aerogel, a hierarchical porous framework with a high specific surface area is formed, featuring efficient mass transfer and ultra-multiple loading sites. The graphene framework enhances visible-light absorption by optimizing TiO_2_ loading, agglomeration behavior and addressing detachable defects through a metal–polyphenol network. After 60 min of illumination, the degradation efficiency exceeds 99.5%, demonstrating superior cycling stability. After 100 cycles, the photocatalytic efficiency remains above 97%, showcasing excellent durability. Furthermore, the in situ polymerized thermoresponsive poly (N-isopropylacrylamide) (PNIPAm) composite exhibits smart responsiveness, enabling reversible temperature-responsive adsorption–desorption behavior within PNIPAm’s LCST range. with an adsorption capacity of 28,000 mg/g at LCST. Heating above LCST desorbs 90.2% of the wastewater, and adsorption stability remains above 98% after 100 thermal cycles, resolving operational challenges in mechanical wastewater recovery. The synergistic integration of an anisotropic porous structure, stable TiO_2_ loading, and thermal responsiveness provides an efficient platform for integrated adsorption and recovery.

## 1. Introduction

Organic dye pollution has been regarded as one of the most critical challenges in water environment remediation [1]. Cationic dyes such as methylene blue (MB) have been extensively used in textile dyeing, papermaking, and chemical industries [2,3,4]. Owing to their conjugated aromatic structures [5] and charged functional groups [6], these dyes exhibit high water solubility and chemical stability [7], rendering them resistant to removal by conventional biological treatment processes [8,9]. Their persistence in aquatic systems not only increases water color and reduces light penetration [10,11], thereby inhibiting photosynthesis, but also poses potential ecological and health risks [12,13,14,15]. Consequently, the development of efficient and sustainable technologies for dye removal has attracted increasing attention.

Photocatalytic oxidation has been considered a promising advanced treatment strategy because it enables the mineralization [16] of organic dyes into CO_2_ and H_2_O [17] under mild conditions. Among various photocatalysts, titanium dioxide (TiO_2_) has been widely investigated due to its chemical stability [18], non-toxicity [19], and strong oxidative capability [20]. Since the pioneering work of Fujishima and Honda in 1972, extensive efforts have been devoted to improving TiO_2_-based photocatalysis [21]. However, its practical application has been severely constrained by the rapid recombination of photogenerated electron–hole pairs [22] and its limited response to visible light, which significantly reduces photocatalytic efficiency under solar irradiation.

To address these limitations, numerous modification strategies have been proposed. Among them, sensitization has been widely adopted because it effectively extends the photoresponse of TiO_2_ into the visible-light [22] region without introducing additional recombination centers. Various sensitizers, including metals, semiconductors, and carbon-based materials, have been coupled with TiO_2_ to form hybrid photocatalysts [23]. In particular, graphene has attracted considerable interest owing to its high electron mobility [24], large specific surface area, and non-toxic nature. The exceptional electron transport capability of graphene [25] facilitated rapid migration of photogenerated electrons, while its large surface area provided abundant sites for TiO_2_ loading [26] and pollutant adsorption. Moreover, the semi-metallic character of graphene enabled it to act as an effective photosensitizer, contributing to enhanced utilization of longer-wavelength light [27]. Related derivatives such as graphene oxide (GO) and graphene-based hybrid structures have also been employed to further improve visible-light photocatalytic performance.

Graphene oxide, featuring abundant oxygen-containing functional groups [28] and high specific surface area, has been widely regarded as an ideal precursor for constructing three-dimensional graphene aerogels (GAs). GAs integrated the intrinsic electronic properties of graphene with a macroscopic, porous, and lightweight architecture, forming interconnected three-dimensional networks. Such structures have been demonstrated to act as efficient electron sinks and fast electron transport pathways [29], thereby suppressing electron–hole recombination in TiO_2_-based systems [30]. Simultaneously, the high porosity and large surface area of GAs enable effective adsorption and enrichment of dye molecules, promoting a synergistic “adsorption–photocatalysis” process. In addition, the monolithic structure of aerogels allows facile separation from water, improving recyclability and reuse.

Nevertheless, conventional isotropic graphene aerogels often suffer from non-uniform pore size distributions [31], limited and unstable TiO_2_ anchoring sites, and severe nanoparticle aggregation. The disordered pore architecture restricts mass transport and hinders dye penetration into the interior of the aerogel, leading to reduced catalytic efficiency. In contrast, anisotropic graphene aerogels (AGA) with highly oriented pore channels have demonstrated superior mass transfer characteristics and structural stability [32]. Directional freeze-casting enables the formation of long-range aligned channels, significantly reducing diffusion resistance and facilitating the rapid transport of dye molecules to active catalytic sites. Furthermore, the oriented three-dimensional porous framework enhances light utilization by inducing multiple scattering and reflection within the aerogel, thereby increasing the effective optical path length and photon harvesting efficiency. The anisotropic structure also provides improved mechanical integrity, allowing the aerogel to maintain structural stability during repeated use [33].

In this study, anisotropic graphene aerogels were fabricated via a combination of hydrothermal reduction in GO [34] and directional freeze-casting, followed by impregnation-based loading of TiO_2_. This mild loading strategy preserved the oriented pore architecture and maximized mass transfer and light utilization advantages. Moreover, a thermos-responsive poly (N-isopropylacrylamide) (PNIPAm)/graphene composite aerogel [35] was constructed, exhibiting spontaneous adsorption at temperatures below 32 °C and autonomous release above the critical temperature. This temperature-triggered adsorption–release behavior enabled a switchable process driven solely by thermal stimuli. Compared with conventional mechanically regenerated graphene aerogels, the proposed system avoided structural damage, reduced energy consumption, and provided a programmable, non-contact regeneration strategy. This work therefore advanced anisotropic graphene aerogels from passive, mechanically regenerated materials toward active, stimuli-responsive platforms for intelligent wastewater treatment.

## 2. Materials and Methods

### 2.1. Materials

Graphite powder (45 μm) was purchased from (Beijing China) Kuer Chemical Co., Ltd. Concentrated sulfuric acid (H_2_SO_4_, 98%), hydrogen peroxide (H_2_O_2_, 30%), potassium permanganate (KMnO_4_), and hydrochloric acid (HCl, 37%) were obtained from (Shanghai China) Sinopharm Chemical Reagent Co., Ltd. L-ascorbic acid, tannic acid, anhydrous ferric chloride (FeCl_3_), titanium dioxide (TiO_2_), methylene blue (MB) and N,N′-methylenebisacrylamide were purchased from (Shanghai, China) Shanghai Aladdin Biochemical Technology Co., Ltd. N-isopropylacrylamide was obtained from (Beijing China) Beijing Inno Chem Science and Technology Co., Ltd., and ammonium persulfate was supplied by (Shanghai China) Sinopharm Chemical Reagent Co., Ltd. All chemicals were of analytical grade and were used as received without further purification unless otherwise specified. Deionized water was used in all experiments.

### 2.2. Preparation of GO

GO was synthesized via a modified Hummers’ method [36,37]. In a typical procedure, graphite powder was added to concentrated sulfuric acid, followed by the gradual addition of the strong oxidizing agent potassium permanganate (KMnO_4_). The mixture was stirred in an ice bath for 2 h to maintain a low reaction temperature and then heated to 55 °C for 6 h to complete the oxidation process. After the reaction, the mixture was slowly poured into ice-cold deionized water to quench the reaction. Once cooled to room temperature, an appropriate amount of hydrogen peroxide (H_2_O_2_) was added until gas evolution ceased, during which the solution color gradually changed from dark brown to yellow. The suspension was then allowed to stand for 12 h to precipitate the solid product. The supernatant was removed, and the remaining solid was repeatedly washed with diluted hydrochloric acid to eliminate residual sulfate ions, followed by thorough rinsing with deionized water until a neutral pH was achieved. The resulting brown dispersion with good fluidity, obtained during the centrifugation and washing process, was collected as GO.

### 2.3. Preparation of AGA

To construct AGA with a highly oriented structure, a mild solution mixing directional freezing strategy was employed. This approach induced the ordered alignment of GO sheets through a directional freezing process prior to chemical reduction, thereby preserving the structural integrity and achieving a well-organized microarchitecture during the subsequent reduction stage. First, an aqueous GO dispersion with a concentration of 4 mg mL^−1^ was prepared. L-ascorbic acid (160 mg) was added to the dispersion and mixed uniformly under magnetic stirring. The resulting dispersion was then sealed in a 20 mL glass vial and placed on the top of a copper rod immersed in a liquid nitrogen/ethanol mixed bath (Self-made in the laboratory). The freezing temperature was precisely controlled by adjusting the volume ratio of liquid nitrogen to ethanol; for example, a liquid nitrogen/ethanol ratio of 1:1 corresponded to −50 °C, 1:2 to −80 °C, 1:4 to −100 °C, and 2:1 to −20 °C. AGA were prepared using a directed freezing process based on an ice-templating strategy. A stable temperature of approximately −50 °C was established using a liquid nitrogen/ethanol cooling bath, thereby creating a unidirectional temperature gradient. Under these conditions, ice crystals preferentially nucleate and grow along the direction of heat flow, whilst graphene oxide flakes and other solutes are displaced and confined between the advancing ice fronts. This process of solute displacement and localized assembly facilitates the formation of an oriented layered structure. Following freeze-drying to remove the ice template, a highly anisotropic porous structure was ultimately obtained, featuring continuous channels along the direction of freezing. During the freezing process, the GO sheets were expelled and aligned along the direction of ice crystal growth, leading to an ordered arrangement. After complete freezing, a relatively stable alignment of GO sheets was formed inside the glass vial. The frozen sample was subsequently transferred to an oven and hydrothermally reduced at 60 °C for 5 h to obtain an anisotropic graphene hydrogel. The hydrogel was then immersed in a 10% ethanol aqueous solution overnight. Finally, anisotropic graphene aerogels were obtained after freeze-drying for 24 h and were denoted as AGA.

### 2.4. Preparation of TPAGA

A robust metal–phenolic network (MPN) was constructed on the as-prepared AGA to serve as an adhesive interlayer for firmly anchoring TiO_2_ particles and improving their immobilization stability on the AGA framework. Briefly, the AGA was first immersed in deionized water to remove residual impurities. The cleaned AGA was then soaked in a tannic acid (TA) solution (1 mg/mL) for 20 min, followed by rinsing with DI water to remove physically adsorbed TA. Subsequently, the TA-treated AGA was immersed in an FeCl_3_ solution (0.5 mg/mL) for 20 min to form a TA-Fe^3+^ coordination network. The sample was thoroughly washed with deionized water to remove excess, uncoordinated Fe^3+^ ions.

Thereafter, a prescribed amount of TiO_2_ powder was added to deionized water and ultrasonicated to obtain a homogeneous TiO_2_ suspension. The MPN-modified AGA was immersed in the TiO_2_ suspension and subjected to mild vacuum infiltration for 10 min to facilitate penetration and diffusion of TiO_2_ into the porous network. After an additional 30 min of immersion, the aerogel was taken out and rinsed with deionized water to remove loosely attached TiO_2_ particles, followed by freeze-drying for 24 h to yield the TiO_2_-loaded AGA.

**Figure 1:**
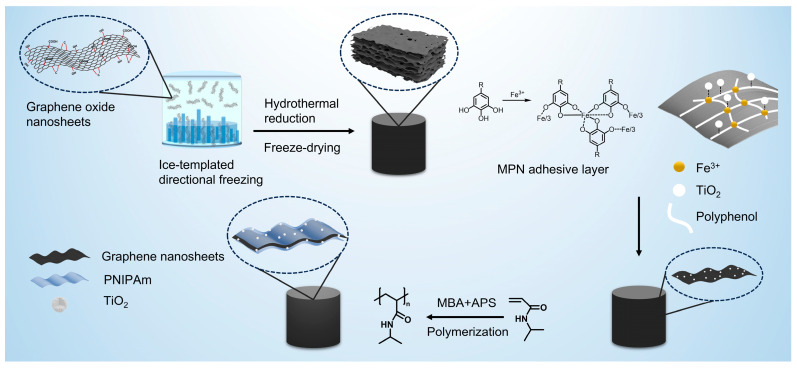
Preparation procedure of TPAGA_(x:y)_.

In the second step, 0.98 g N-isopropylacrylamide (NIPAm), 0.05 g N,N′-methylenebisacrylamide (MBA), and ammonium persulfate (APS) were dispersed in 20 mL deionized water. The amount of APS added was 1% of the monomer weight. By adding an MBA initiator equivalent to 1% of the monomer weight, the cross-linking density was controlled, resulting in the preparation of PNIPAm with medium to low cross-linking. Prior to polymerisation, the reaction system was purged with nitrogen for 15 min to remove dissolved oxygen and prevent inhibition of radical polymerisation. Subsequently, the TiO_2_-loaded AGA was immersed in the solution and reacted at 60 °C for 8 h to ensure complete conversion of the monomer and the formation of a stable PNIPAm network structure within the aerogel matrix. After completion, excess PNIPAm on the outer surface was carefully removed, and the resulting composite aerogel was freeze-dried for 24 h to obtain the TiO_2_-loaded PNIPAm-integrated graphene aerogel (TPAGA). Based on the TiO_2_ loading concentration, the composite aerogels were named TPAGA_(4:1)_, TPAGA_(2:1)_, TPAGA_(1:1)_, and TPAGA_(1:2)_ (hereinafter referred to as TPAGA_(x:y)_, where (x:y) represents the mass ratio of TiO_2_ to GO). The preparation procedure of TPAGA_(x:y)_ is shown in Figure 1. The proportions of the various components are shown in Table 1.

### 2.5. Characterization

Fourier transform infrared (FTIR) spectroscopy was performed on a Thermo Fisher Scientific (Delaware, United States) Nicolet IS10 spectrometer over a wavenumber range of 400–4000 cm^−1^. The crystalline structure of the aerogels was analyzed using X-ray diffraction (XRD) in the 2θ range of 5–90°, employing a Rigaku (Tokyo, Japan) D/MAX/2500 PC diffractometer. Raman spectra were collected with a laser excitation wavelength of 532 nm. X-ray photoelectron spectroscopy (XPS) was conducted on a Thermo Fisher Scientific (Delaware, United States) ESCALAB XI+ instrument to analyze the surface elemental composition and chemical states. The morphologies and structures of the aerogel were observed by scanning electron microscope (SEM, Hitachi regulus8100, Tokyo, Japan) and energy dispersive spectrometer (EDS, Tokyo, Japan). The specific surface area, pore size, and pore size distribution were determined using a BET surface area and porosity analyzer (TB400, Beijing China). The concentration of methylene blue (MB) in aqueous solution was determined using UV-visible spectroscopy (UV-vis, Tokyo, Japan).

### 2.6. Adsorption Capacity and Recyclability

The MB aqueous solution was adsorbed at room temperature. The adsorption capacity (*Q*) of the aerogel was determined by weighing the aerogel before and after adsorption equilibrium. The adsorption capacity was calculated according to the following equation:

(1) $$Q=\frac{m_{2}-m_{1}}{m_{1}}$$

where $m_{1}$ and $m_{2}$ represent the mass of aerogels before and after saturation, respectively.

The treated wastewater was recovered by increasing the temperature to 45 °C. After the aerogel reached adsorption saturation and completed photodegradation at room temperature, the temperature was raised to induce autonomous dehydration. The aerogel was allowed to release water until its weight no longer changed, after which it was weighed. One hundred consecutive cycling tests were conducted to evaluate the photocatalytic degradation of the MB solution and the temperature-stimulated responsive behavior of TPAGA. The photocatalytic degradation efficiency (*D*) and adsorption stability (*S*) of TPAGA were calculated as follows:

(2) $$D=\frac{A_{0}-A_{t}}{A_{0}}\times 100\%$$

(3) $$S=1-\frac{m_{max}-m_{min}}{m_{1}}\times 100\%$$

where *A*_0_ is the absorbance after dark adsorption equilibrium, *A_t_* is the absorbance after light irradiation for time (*t*), and the calculation is based on the Beer–Lambert law; $m_{max}$ and $m_{min}$ represent the weights of the aerogel at maximum and minimum adsorption, $m_{1}$ is the weight of the aerogel before adsorption.

## 3. Results and Discussion

### 3.1. Structural Characterization

**Figure 2:**
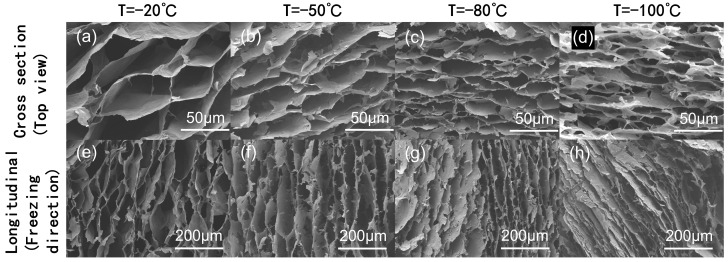
SEM images of the porous network formed by AGA (4 mg/mL GO) at different freezing temperatures; (**a**–**d**) cross-sectional and (**e**–**h**) longitudinal sectional pore structures at substrate temperatures of −20 °C, −50 °C, −80 °C, and −100 °C.

It was illustrated in Figure 2 that the structural characteristics of AGA were prepared via thermal–chemical reduction. As shown in Figure 2a–d, the cross-sectional images reveal typical honeycomb-like macroporous architectures. The longitudinal sections, Figure 2e–h, demonstrate interconnected channels throughout the network. SEM imaging enabled analysis of the aerogel macrostructure and average pore size. At a freezing temperature of −20 °C, the average pore size reached 147 μm with uniform distribution, attributed to the relatively slow ice crystal growth that allowed graphene sheets to align orderly along the temperature gradient, forming well-oriented channel structures. As the freezing temperature decreased to −50 °C, the pore size shrank to approximately 72 μm, accompanied by an increase in pore number. Further lowering to −80 °C resulted in an average pore size of 54 μm and a more irregular distribution, where rapid ice crystal growth caused partial compression of the graphene layers and the formation of numerous fine pores. Despite this, the directionality of the channels was preserved due to the precisely controlled temperature gradient. At −100 °C, the average pore size was further reduced to ~23 μm, and stacked lamellar structures became more apparent. Nevertheless, a clear honeycomb-like anisotropic architecture was still evident, indicating the robustness of the directional freezing method in maintaining orientation even under extreme conditions.

Temperature is also a key factor influencing the pore structure of aerogels during the directed freezing process. According to the ice-template mechanism, at lower temperatures, the nucleation rate of ice crystals is faster, resulting in a pore structure with smaller, uniformly distributed pores; whereas at higher temperatures, ice crystals grow more extensively, tending to form larger pores. This difference further affects the specific surface area and mass transfer behavior, thereby exerting a certain influence on adsorption and photocatalytic performance. In this study, the freezing temperature was kept constant to highlight the influence of reduction time on structure and performance. In contrast, reduction time exerts a more direct regulatory effect on the restoration of the sp^2^ structure of graphene and its electron transport capability and therefore plays a dominant role in photocatalytic performance.

**Figure 3:**
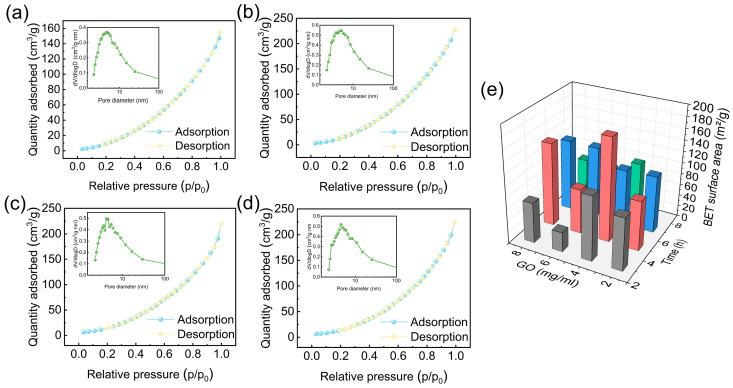
BET surface area analysis and pore size distribution plots of AGA prepared with a GO loading of 4 mg/mL; chemical–thermal reduction times of (**a**) 3 h; (**b**) 5 h; (**c**) 7 h; (**d**) 9 h; (**e**) comprehensive analysis of AGA prepared with GO loadings of 2–4 mg/mL and reduction times of 3–9 h.

**Figure 4:**
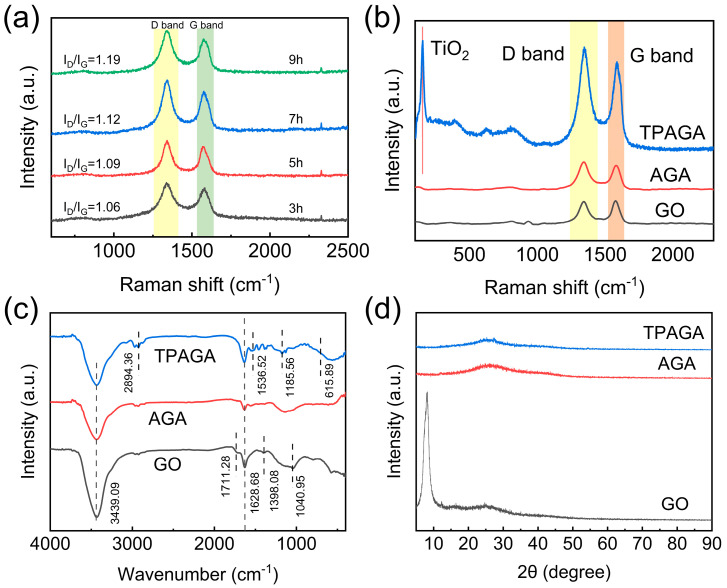
(**a**) Raman spectrum of AGA; (**b**) Raman spectrum of GO, AGA and TPAGA; (**c**) FTIR spectrum of GO, AGA and TPAGA; (**d**) XRD spectrum of GO, AGA and TPAGA.

The BET isotherms and pore size distributions of AGA (GO = 4 mg/mL) reduced for different durations (3, 5, 7, and 9 h) are further presented in Figure 3. All samples exhibited typical type III, indicating the presence of mesoporous structures formed by stacked graphene sheets. As the reduction time increased from 3 h to 5 h, the adsorption volume rose markedly, and the specific surface area increased from 112.34 m^2^/g to 179.62 m^2^/g. The pore size distribution became more concentrated within the 7–10 nm mesoporous range, suggesting that oxygen-containing groups were partially removed, and the framework became more stable with the highest porosity. When the reaction time was further extended to 7 h and 9 h, the adsorption volume and specific surface area (124.75 m^2^/g and 82.49 m^2^/g, respectively) decreased significantly, while the pore size distribution broadened and shifted toward larger pores due to partial pore collapse and blockage. SEM observations corroborated the BET results: the 5 h sample displayed well-aligned lamellar structures with intact pore walls, whereas the 7 h and 9 h samples showed structural shrinkage and increased stacking, leading to reduced pore connectivity. Figure 4a presents the Raman spectra of AGA prepared at different reduction times. All spectra exhibited two characteristic peaks located at approximately 1350 cm^−1^ and 1580 cm^−1^, which were assigned to the D band and G band, respectively. The degree of structural disorder was evaluated using the intensity ratio of the D band to the G band (I_D_/I_G_), where a higher ratio indicates a higher density of structural defects. As the reduction time increased, the I_D_/I_G_ value gradually increased from 1.06 for 3 h to 1.19 for 9 h, indicating a strong correlation between the reduction degree and the structural disorder of the aerogel [38]. However, excessive structural disorder is not necessarily beneficial for subsequent functionalization and photocatalytic performance. An overly high defect density may disrupt the continuity of the graphene conductive network, leading to increased charge recombination and hindered electron transport across the aerogel framework.

In summary, a moderate thermal reduction time (approximately 5 h) was favorable for constructing a stable mesoporous aerogel architecture with a high specific surface area, which is critical for photocatalytic applications. In contrast, insufficient reduction led to structural instability, whereas excessive reduction resulted in excessive densification and loss of accessible porosity. Moreover, prolonged reduction induced an increased density of structural defects in the aerogel framework, which was detrimental to subsequent TiO_2_ loading and the in situ polymerization of PNIPAm, thereby impairing interfacial charge transfer. The high specific surface area of AGA provided abundant accessible active sites and enlarged interfacial contact areas, effectively promoting the adsorption of reactants, facilitating the separation and migration of photogenerated charge carriers, and enhancing interfacial electron transfer efficiency, ultimately contributing to improved photocatalytic performance.

The superior interfacial properties of AGA stem from their oriented channel structure, which differs significantly from isotropic, randomly interconnected pore structures. These oriented channels reduce mass transfer resistance, enabling rapid axial diffusion of reactants and products, thereby enhancing the accessibility of active sites; in contrast, isotropic structures, due to their highly tortuous pore networks, are prone to diffusion limitations, which hinder the efficient progression of interfacial reactions. Furthermore, the anisotropic framework facilitates the uniform distribution of TiO_2_ nanoparticles and MPN along the channel walls, reducing agglomeration and enhancing interfacial contact, thereby improving the transport efficiency of photo-generated carriers and the utilization of active sites. These structural advantages collectively contribute to improvements in adsorption kinetics, photocatalytic efficiency and cycle stability, demonstrating superior interfacial performance.

In Figure 4b, Raman spectra of GO, AGA, and TPAGA were compared to further elucidate the structural integrity and component incorporation. GO exhibited relatively weak D and G bands, while both AGA and TPAGA showed pronounced D and G bands, confirming the preservation of the sp^2^ carbon framework after aerogel formation and subsequent functionalization. Notably, TPAGA displayed an additional low-frequency Raman signal around ~200 cm^−1^, which was attributed to Ti–O lattice vibrations, providing direct evidence for the successful incorporation of TiO_2_. Meanwhile, the enhanced D band intensity of TPAGA indicated increased interfacial defects arising from the anchoring of the metal–polyphenol network, TiO_2_ loading, and in situ polymerization of PNIPAm, rather than the destruction of the graphene skeleton. The chemical structure evolution was further confirmed by FTIR spectra Figure 4c. GO exhibited typical absorption bands associated with oxygen-containing functional groups, including a broad O–H stretching band at ~3439 cm^−1^, a C=O stretching vibration at ~1711 cm^−1^, and C–O related bands in the range of 1200–1000 cm^−1^. After thermal reduction and aerogel formation, these oxygen-related peaks were significantly weakened in AGA, indicating partial deoxygenation and restoration of the graphene framework. In contrast, TPAGA displayed newly enhanced absorption bands at ~1650 cm^−1^ and ~1536 cm^−1^, corresponding to the amide I and amide II bands, respectively, confirming the successful in situ polymerization of N-isopropylacrylamide. Additionally, the intensified broad band in the 3300–3500 cm^−1^ region was attributed to the overlapping contributions of N–H stretching vibrations and hydrogen-bonded hydroxyl groups from the metal–polyphenol network, suggesting the formation of a hydrogen-bond-rich interfacial architecture. The XRD patterns shown in Figure 4d further supported the structural transformation. GO exhibited a sharp diffraction peak at approximately 10°, corresponding to the (001) plane of oxidized graphene layers. This peak disappeared completely after aerogel formation, while a broad diffraction feature centered at ~24–26° emerged in AGA and TPAGA, indicative of a disordered multilayer graphene structure with low crystallinity. No distinct diffraction peaks associated with crystalline TiO_2_ were observed in TPAGA, which could be attributed to the nanoscale dispersion, and strong interfacial interactions between TiO_2_, the metal–polyphenol network, and the graphene aerogel matrix.

**Figure 5:**
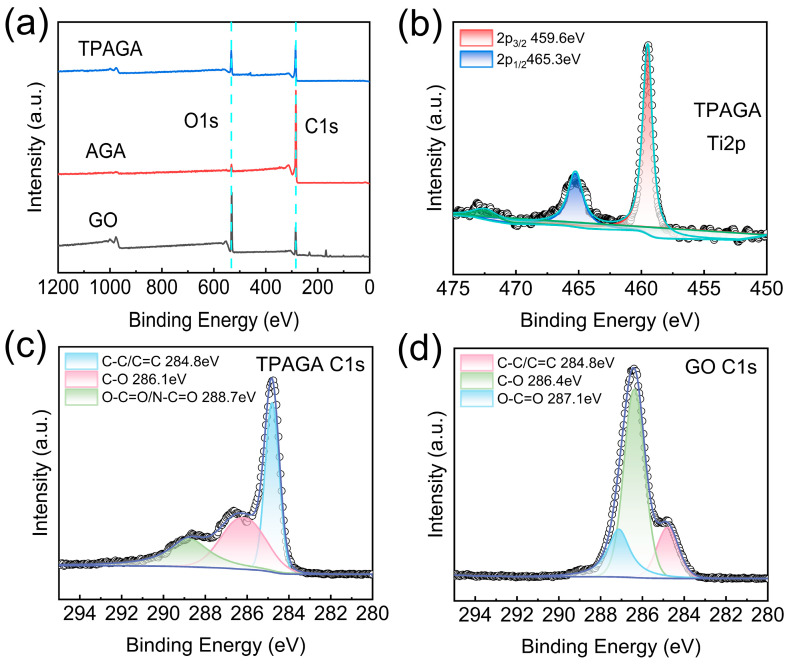
(**a**) XPS spectrum of GO, AGA and TPAGA; XPS spectrum of TPAGA; (**b**) Ti 2p spectrum; (**c**) C 1s spectrum; (**d**) XPS spectrum of GO C 1s.

To further elucidate the surface chemical composition and interfacial interactions of the TPAGA composite, X-ray photoelectron spectroscopy (XPS) was employed, as shown in Figure 5. As presented in Figure 5a, the survey spectra of GO, AGA, and TPAGA revealed the presence of C and O elements in all samples. Compared with GO, the O 1s signal intensity of AGA decreased markedly, indicating partial removal of oxygen-containing functional groups during the thermal reduction process, which was consistent with the FTIR and XRD results. Notably, TPAGA exhibited a relatively enhanced O 1s signal intensity compared with AGA, which could be attributed to the introduction of oxygen-rich components, including the metal–polyphenol network and TiO_2_, as well as the incorporation of PNIPAm containing amide functionalities. The high-resolution Ti 2p spectrum of TPAGA, Figure 5b, displayed two well-defined peaks located at approximately 459.6 eV and 465.3 eV, corresponding to Ti 2p_3/2_ and Ti 2p_1/2_, respectively. The characteristic spin–orbit splitting and binding energies were in good agreement with those reported for Ti^4+^ species in TiO_2_, confirming that TiO_2_ was successfully introduced into the TPAGA framework while maintaining its chemical state. The absence of additional Ti-related components further suggested that TiO_2_ was stably anchored within the composite without significant phase transformation. The chemical states of carbon were further analyzed by deconvolution of the high-resolution C 1s spectrum of TPAGA Figure 5c. Three main components were identified at 284.8 eV, 286.1 eV, and 288.7 eV, which were assigned to C–C/C=C, C–O, and O–C=O/N–C=O species, respectively. Compared with GO Figure 5d, TPAGA exhibited a pronounced decrease in oxygenated carbon species associated with highly oxidized groups, accompanied by the emergence and enhancement of the high-binding-energy component at ~288.7 eV. This component was attributed not only to residual carboxyl groups but also to the amide carbonyl groups originating from the in situ polymerization of PNIPAm, providing strong evidence for the successful incorporation of the thermoresponsive polymer. Compared with graphene oxide, the enhanced intensity of the C–C/C=C peak in TPAGA can be attributed to the partial reduction in graphene oxide during the synthesis process, which leads to the removal of oxygen-containing functional groups and the restoration of sp^2^ carbon regions. Although PNIPAm introduces C–N and N–C=O functional groups, its contribution to the overall C 1s spectrum is relatively limited due to its low content and its interpenetrating distribution within the aerogel framework. Furthermore, the formation of a metal–polyphenol network and the anchoring of TiO_2_ nanoparticles further alter the surface chemical environment, leading to a relative enhancement of the graphitic carbon signal. Consequently, the observed spectral evolution reflects the dual effects of graphene oxide reduction and the formation of the composite structure. In contrast, the C 1s spectrum of GO Figure 5d was dominated by oxygen-containing functional groups, including C–O (~286.4 eV) and O–C=O (~287.1 eV), reflecting its highly oxidized nature. The clear differences between the C 1s spectra of GO and TPAGA further confirmed the structural transformation from oxidized graphene sheets to a hierarchically functionalized aerogel system. Collectively, the XPS results corroborated the successful construction of TPAGA through the integration of a reduced graphene–aerogel skeleton, a TA–Fe^3+^ metal–polyphenol interfacial network, TiO_2_ nanoparticles, and in situ polymerized PNIPAm. During the preparation of AGA, the carbon-to-oxygen ratio of GO increased from 0.51 to 4.92 following chemical reduction (Table 2). After loading titanium dioxide, a strong characteristic peak for Ti appeared, and the characteristic peak for O 1s significantly increased in TPAGA_(1:1)_, indicating successful loading of TiO_2_ and successful polymerization of PNIPAm. These surface chemical features are expected to facilitate strong interfacial interactions and efficient charge transfer, thereby supporting the enhanced photocatalytic performance observed in subsequent experiments. In this system, MPN not only serves as a surface modification layer but, more importantly, acts as an interfacial adhesion layer linking the graphene framework to the TiO_2_ nanoparticles. This network is formed through coordination and complexation between the phenolic hydroxyl groups in TA molecules and Fe^3+^, constructing a cross-linked Fe–O coordination structure. As TA molecules contain both aromatic structures and abundant hydroxyl sites, the resulting MPN can be firmly adsorbed onto the surface of the graphene–aerogel framework via π–π interactions and hydrogen bonding, thereby forming a continuous coating on the outer layer of the framework. Furthermore, as MPNs are rich in oxygen-containing functional groups and the TiO_2_ surface typically exhibits hydroxylation, further hydrogen bonding, surface complexation and coordination interactions can occur between the two, thereby effectively anchoring the TiO_2_ nanoparticles to the scaffold surface. Consequently, the MPN effectively acts as an ‘interfacial bridge’ within the system, not only improving the uniformity of TiO_2_ dispersion but also suppressing particle agglomeration and detachment during circulation, whilst enhancing interfacial contact and charge transport efficiency between the TiO_2_ and the conductive graphene scaffold.

**Figure 6:**
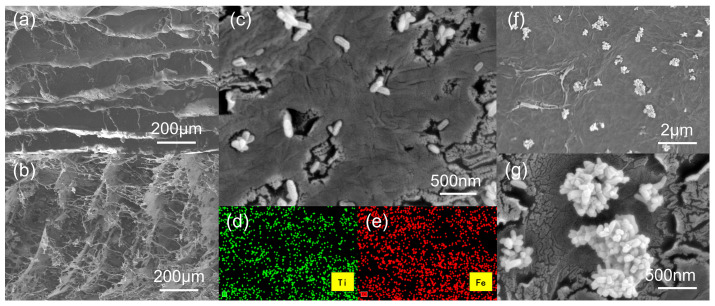
(**a**,**b**) Cross-sectional and longitudinal SEM images of TPAGA; (**c**) SEM image of TPAGA after low TiO_2_ loading (GO/TiO_2_ = 1/1); EDS mapping of TPAGA (**d**) Ti element, (**e**) Fe element; (**f**) and (**g**) SEM image of TPAGA after high TiO_2_ loading (GO/TiO_2_ = 1/2).

Following the structural and chemical characterizations discussed in Figure 4 and Figure 5, the morphology and elemental distribution of TPAGA were further investigated by SEM and EDS mapping, as shown in Figure 6, to elucidate the hierarchical architecture and spatial dispersion of functional components. As displayed in Figure 6a,b, the aerogel skeleton maintained structural integrity without noticeable collapse, indicating that the subsequent introduction of the metal–polyphenol network, TiO_2_, and PNIPAm did not compromise the macroscopic architecture of AGA. The higher-magnification SEM image in Figure 6c further illustrated the surface morphology of TPAGA after low TiO_2_ loading. Discrete nanoscale particles were clearly observed to be uniformly anchored on the aerogel skeleton, without severe aggregation. These particles were attributed to TiO_2_, whose homogeneous distribution can be reasonably associated with the strong interfacial affinity provided by the TA–Fe^3+^ metal–polyphenol network, acting as an effective binding layer between the inorganic nanoparticles and the graphene framework. Such uniform anchoring is consistent with the absence of distinct TiO_2_ diffraction peaks in the XRD pattern, Figure 4d, and the well-defined Ti^4+^ chemical states observed in the Ti 2p XPS spectrum, Figure 5b. To further confirm the elemental distribution, EDS mapping analysis was conducted, as shown in Figure 6d,e. The Ti elemental map, Figure 6d, exhibited a homogeneous spatial distribution throughout the aerogel matrix, confirming that TiO_2_ was uniformly dispersed rather than locally aggregated. Meanwhile, the Fe elemental map, Figure 6e, also displayed a uniform distribution, providing direct evidence for the successful formation of a TA–Fe^3+^ metal–polyphenol network that was evenly integrated within the aerogel skeleton. The spatial overlap and uniformity of Ti and Fe signals suggested that the metal–polyphenol network effectively mediated the immobilization of TiO_2_ and promoted strong interfacial coupling among the graphene framework, inorganic nanoparticles, and polymeric components. However, when the TiO_2_ loading was increased, as shown in Figure 6f,g, aggregation of TiO_2_ nanoparticles became apparent. The high TiO_2_ content resulted in the formation of larger TiO_2_ clusters, which were not only less efficient in terms of photocatalytic activity due to limited surface area but also hindered electron transfer due to poor interfacial contact between the TiO_2_ particles and the graphene framework. This aggregation led to the blocking of active sites, reducing the overall photocatalytic efficiency.

Taken together with the Raman, FTIR, XRD, and XPS results discussed in Figure 4 and Figure 5, these morphological and elemental analyses unambiguously demonstrated that TPAGA possessed a hierarchically organized structure featuring an anisotropic graphene aerogel backbone, a uniformly distributed metal–polyphenol interfacial network, and highly dispersed TiO_2_ nanoparticles. This well-integrated architecture provided abundant accessible active sites, enlarged interfacial contact areas, and continuous electron transport pathways, which are crucial for efficient interfacial charge transfer and enhanced photocatalytic performance. In the TPAGA system, PNIPAm is formed within the pre-fabricated porous aerogel scaffold via in situ radical polymerization. Under the conditions of this study, PNIPAm exists primarily as a lightly cross-linked polymer network rather than as discrete microgel particles. The polymer chains are distributed along the inner walls of the pores and within the interconnected pore structure, partially coating the surface of the scaffold whilst maintaining the openness of the overall anisotropic porous structure.

The polymer content and cross-linking density are controlled by the initial ratio of NIPAm to MBA, and optimization is employed to prevent pore blockage caused by excess polymer. In terms of interaction mechanisms, PNIPAm does not undergo covalent grafting onto the graphene scaffold, but rather exists within the aerogel via physical confinement, achieving stable fixation through hydrogen bonding and interfacial interactions with the MPN. This structure ensures both the structural stability of the material and endows it with temperature-responsive, reversible expansion–contraction properties.

### 3.2. Photocatalytic Degradation Performance of TPAGA

In order to evaluate the performance of the composite materials more accurately, a clear control system was established in this study. Firstly, AGA was used as a skeletal control to distinguish between the contributions of the porous graphene framework itself and those resulting from the introduction of functional groups; secondly, TPAGA samples with different GO/TiO_2_ ratios were systematically compared to elucidate the influence of TiO_2_ loading on adsorption behavior, photocatalytic degradation efficiency and cycling stability. Through these comparisons, the specific roles of each structural component can be more clearly identified, and the optimal composition ratio determined.

**Figure 7:**
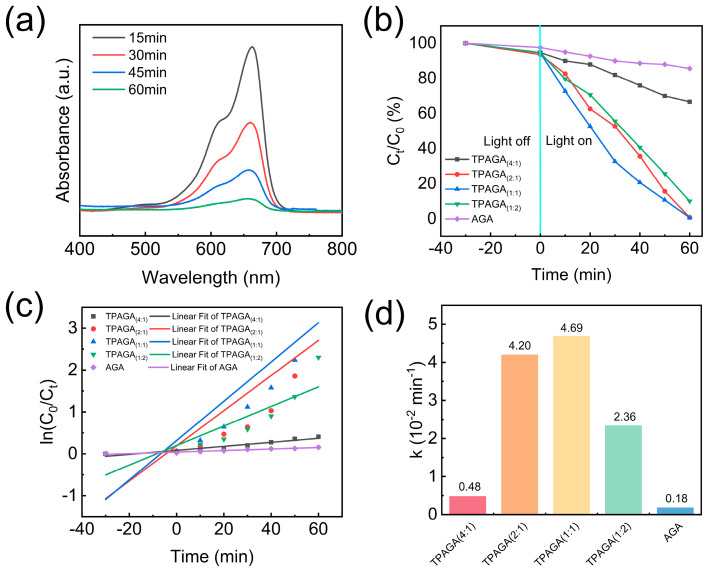
UV–vis absorption spectra of TPAGA_(1:1)_ during photocatalytic degradation of MB solutions at an initial concentration of 50 mg/L; (**a**) photocatalytic performance of TPAGA_(x:y)_ in degrading MB under visible-light irradiation; (**b**) concentration decay curve; (**c**) pseudo-first-order kinetic curve; (**d**) reaction rate constant.

The photocatalytic degradation performance of TPAGA with different TiO_2_ loadings was evaluated by monitoring the UV–vis absorption spectra of MB solutions with an initial concentration of 50 mg/L, as shown in Figure 7. The characteristic absorption peak of MB at approximately 664 nm was recorded at different irradiation times (15, 30, 45, and 60 min) to assess the degradation behavior. To distinguish the effects of adsorption and photocatalysis on MB degradation, the concentration decay curves before and after illumination were monitored (Figure 7b). After 30 min of dark adsorption, MB concentrations in all samples decreased only slightly, attributable to physical adsorption and surface enrichment. Following visible-light irradiation, the concentration decay rate of TPAGA_(x:y)_ samples was significantly faster than that of pristine AGA, confirming photocatalysis as the dominant mechanism in MB degradation. Among them, the TPAGA_(1:1)_ sample exhibited the fastest degradation rate, with MB concentration reduced to only 0.66% of the initial level after 60 min. In contrast, the AGA sample showed negligible degradation after 60 min of irradiation, retaining 85.66% of the initial concentration, highlighting the crucial role of TiO_2_ incorporation. Further analysis of the photocatalytic kinetics was conducted using the pseudo-first-order kinetic model, ln(C_0_/C_t_) = kt, to fit the photocatalytic degradation data. As shown in Figure 7c, all samples exhibited a good linear relationship between ln(C_0_/C_t_) and irradiation time, indicating that MB degradation follows pseudo-first-order reaction kinetics. The apparent reaction rate constants (k) were extracted from the slopes of the fitted curves and summarized in Figure 7d. TPAGA_(1:1)_ exhibited the highest k value of 4.69, significantly higher than other samples. This result indicates that the optimal TiO_2_ loading ratio is 1:1, which provides sufficient photocatalytic active sites while avoiding nanoparticle aggregation caused by excessive TiO_2_ loading, thereby preventing light penetration and mass transport hindrance. The outstanding photocatalytic performance of TPAGA_(1:1)_ can be attributed to the synergistic effect between its anisotropic porous structure and graphene. Highly oriented layered channels facilitate rapid solution transport, enhancing interfacial contact between methylene blue molecules and photocatalytic sites. Concurrently, the graphene framework promotes charge migration while suppressing hole recombination. Thus, the integration of anisotropic architecture with TiO_2_ loaded onto an MPN achieves efficient and stable photocatalytic degradation of organic dyes, demonstrating TPAGA composite materials’ application potential in wastewater treatment.

Overall, the photocatalytic degradation efficiency of TPAGA exhibits a pronounced dependence on TiO_2_ loading, following a volcano-shaped trend. The optimal TiO_2_ loading (TiO_2_:GO = 1:1) must be achieved to balance the number of active sites with interfacial charge transfer efficiency. Excessive TiO_2_ loading leads to nanoparticle aggregation, impairing interfacial electron transport and significantly reducing photocatalytic performance. These findings are highly consistent with the structural and morphological analyses shown in Figure 4, Figure 5 and Figure 6, highlighting the critical role of TiO_2_ dispersion in achieving efficient photocatalytic degradation. During the photocatalytic process, reactive oxygen species such as hydroxyl radicals (·OH) and superoxide radicals (·O_2_^−^) are generated; these species may affect the organic components and interfacial structures within the composite material. In this system, TiO_2_ exhibits good chemical stability and is unlikely to undergo structural changes under photocatalytic conditions. The MPN formed by TA and Fe^3+^ constructs a stable structure through strong coordination, endowing it with a high resistance to chemical disturbances. As for PNIPAm, although it may degrade under strong oxidative conditions, in this system the polymer network is confined by the aerogel scaffold and partially protected by the graphene structure and the MPN layer, thereby reducing direct attack by reactive species. Furthermore, the relatively mild reaction conditions and limited reaction time in the system further mitigate potential degradation. Combined with the results of cyclic stability tests, this indicates that the composite system exhibits good structural stability and functional retention in a photocatalytic reaction environment.

**Figure 8:**
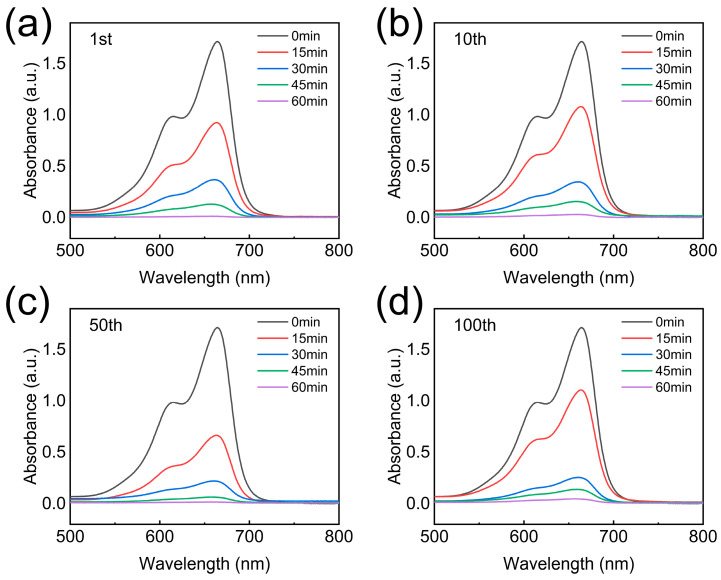
UV–vis absorption spectra of MB (initial concentration: 30 mg/L) during cyclic photocatalytic degradation over TPAGA(GO/TiO_2_ = 1/1): (**a**) 1st cycle, (**b**) 10th cycle, (**c**) 50th cycle, and (**d**) 100th cycle.

Following the evaluation of photocatalytic performance under different TiO_2_ loadings (Figure 7), the photocatalytic stability and reusability of TPAGA_(1:1)_ were further investigated through cyclic degradation experiments using MB solutions with an initial concentration of 30 mg/L. The corresponding UV–vis absorption spectra recorded at different irradiation times during the 1st, 10th, 50th, and 100th cycles are presented in Figure 8. As shown in Figure 8a, during the first photocatalytic cycle, a rapid and continuous decrease in the characteristic MB absorption peak at approximately 664 nm was observed with increasing irradiation time, indicating efficient photocatalytic degradation. After 60 min of irradiation, the absorption peak was almost completely suppressed, demonstrating the high initial photocatalytic activity of TPAGA_(1:1)_. In Figure 8b, corresponding to the 10th cycle, a degradation behavior similar to that of the first cycle was maintained. Only a slight increase in residual absorption intensity was detected, suggesting that the photocatalytic activity was largely preserved after repeated use. This observation indicated that the active sites and interfacial structure of TPAGA_(1:1)_ remained stable during multiple degradation cycles. As the number of cycles increased to 50 (Figure 8c) and 100 (Figure 8d), the degradation efficiency showed a minor but gradual decline. Nevertheless, a substantial reduction in MB absorption intensity was still achieved within 60 min of irradiation, confirming that TPAGA_(1:1)_ retained considerable photocatalytic activity even after prolonged cycling. The absence of significant peak shifts or spectral distortion suggested that no obvious structural degradation or catalyst deactivation occurred during repeated use.

The excellent cyclic stability observed in Figure 8 was closely related to the robust hierarchical architecture of TPAGA. As demonstrated in Figure 4, Figure 5 and Figure 6, the anisotropic graphene–aerogel framework provided mechanical stability and continuous electron transport pathways, while the metal–polyphenol network effectively anchored TiO_2_ nanoparticles and suppressed their detachment or aggregation during cycling. Moreover, the optimized TiO_2_ loading identified in Figure 7 ensured a balance between active site availability and aggregation resistance, thereby contributing to the long-term photocatalytic durability. Overall, the cyclic photocatalytic experiments confirmed that TPAGA exhibited excellent reusability and structural stability, making it a promising photocatalyst for practical wastewater treatment applications.

**Figure 9:**
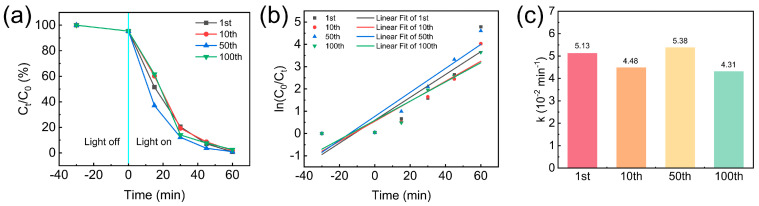
Cyclic stability and kinetic analysis of TPAGA_(1:1)_: (**a**) concentration decay curve during cycling; (**b**) pseudo-first-order kinetic curves in different cycles; (**c**) reaction rate constant.

As shown in Figure 9, the cyclic stability and kinetic fitting curves of TPAGA_(1:1)_ photocatalytically degrade MB under visible-light irradiation. As shown in Figure 9a, during the dark reaction adsorption phase, only a slight decrease in MB concentration was observed, indicating limited dark reaction adsorption. Upon illumination initiation, C_t_/C_0_ exhibited a rapid decreasing trend across all test cycles, confirming the ongoing degradation process. Notably, the degradation curves for cycles 1, 10, 50, and 100 showed high overlap, indicating that TPAGA_(1:1)_ maintained highly stable photocatalytic activity during repeated use. The pseudo-first-order kinetic model was used to fit the photocatalytic degradation data (as shown in Figure 9b). The ratio of lnC_0_/C_t_ exhibited a linear relationship with irradiation time and a high correlation coefficient, indicating that the degradation process consistently followed pseudo-first-order kinetics throughout the cyclic testing. Notably, the slope of the fitted curve showed only minor variations between cycles, suggesting that intrinsic activity loss after long-term operation is negligible. The rate constants (k) extracted from linear fits were summarized in Figure 9c. Over 100 cycles, k consistently remained within the range (4.31–5.38) 10^−2^/min, with no significant decay observed. Such minor fluctuations can be attributed to experimental error or slight surface reconstruction during repeated reactions, rather than a decline in catalytic performance due to structural degradation of TPAGA. The outstanding kinetic stability highlights the strong interfacial coupling between AGA, TiO_2_ nanoparticles, and the metal–polyphenol network. This coupling effectively suppresses TiO_2_ agglomeration, ensuring sustained charge transfer during prolonged photocatalytic operation. These results demonstrated that TPAGA_(1:1)_ possesses outstanding photocatalytic durability and kinetic stability, making it a promising recyclable platform for practical wastewater treatment applications.

### 3.3. Temperature Responsiveness of TPAGA_(1:1)_

**Figure 10:**
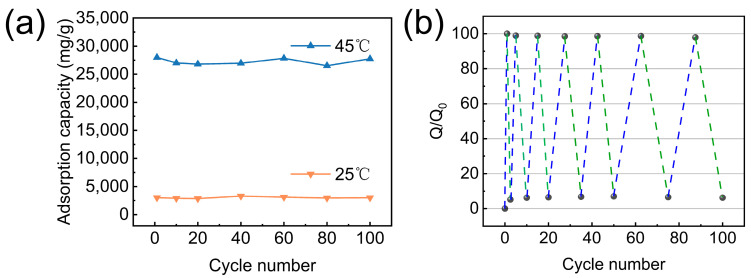
Temperature-responsive adsorption–desorption cycling performance of TPAGA: (**a**) adsorption capacity at 25 °C and 45 °C over 100 consecutive cycles; (**b**) normalized adsorption capacity (Q/Q_0_), blue line represents adsorption, green line represents desorption.

The temperature-responsive adsorption–desorption cycling performance and long-term photocatalytic durability were further evaluated, as summarized in Figure 10. As shown in Figure 10a, the in situ polymerized thermoresponsive PNIPAm composite exhibits smart responsiveness, enabling reversible temperature-responsive adsorption–desorption behavior within PNIPAm’s LCST range, with an adsorption capacity of 28,000 mg/g at LCST. Heating above LCST desorbs 90.2% of the wastewater, and adsorption stability remains above 98% after 100 thermal cycles, confirming the robustness and reversibility of the thermoresponsive adsorption–desorption process. The normalized adsorption capacity (Q/Q_0_) presented in Figure 9b further illustrated the reversible adsorption–desorption behavior during repeated temperature switching. The Q/Q_0_ values exhibited a stable and reproducible oscillation between low and high levels corresponding to adsorption and desorption states, respectively, without noticeable attenuation over 100 cycles. This behavior demonstrated that the temperature-triggered recovery efficiency of TPAGA for wastewater adsorption was well preserved during long-term operation.

The LCST of PNIPAm in TPAGA is regulated by the cross-linking density, following the general principle that an increase in cross-linking density leads to a decrease in LCST [39]. As revealed in the review by Stetsyshyn et al., cross-linking sites restrict the flexibility of PNIPAm chains, thereby inhibiting the formation of hydrogen bonds between amide groups and water molecules at T < LCST. Concurrently, at T > LCST, the cross-linked network brings adjacent PNIPAm segments closer together, promoting intramolecular hydrogen bonding and hydrophobic aggregation, thereby lowering the phase transition temperature. For TPAGA_(1:1)_, a moderate cross-linking density (ν_e_ = 1.86 × 10^3^ mol/m^3^) balances chain flexibility and network stability: the porous structure of the anisotropic graphene aerogel provides ample space for the extension of PNIPAm chains, offsetting the restrictive effect of cross-linking. Consequently, the LCST of PNIPAm in TPAGA remains at approximately 30 °C, ensuring efficient thermoresponsive adsorption–desorption behavior.

The exceptional adsorption capacity of TPAGA composites can be attributed to the synergistic interaction of multiple physicochemical mechanisms, rather than a single adsorption mechanism. Firstly, electrostatic attraction plays a significant role, as there is a strong attraction between the positively charged MB molecules and the negatively charged functional groups on the graphene scaffold and the metal–polyphenol network. Secondly, π–π stacking occurs between the aromatic rings of MB and the sp^2^-hybridized carbon regions restored in the graphene aerogel, thereby enhancing the adsorption affinity. Thirdly, hydrogen bonding between the amide groups of PNIPAm, the phenolic hydroxyl groups in the metal–polyphenol network, and the MB molecules further stabilizes the adsorption process. Furthermore, the thermoresponsive behavior exhibited by PNIPAm below the LCST promotes the formation of a hydration layer, which facilitates dye diffusion and adsorption via a hydration-assisted mechanism. Structurally, the anisotropic porous architecture provides continuous transport pathways and highly accessible active sites, significantly reducing mass transfer resistance. Each component plays a unique role: the graphene aerogel serves as a high-surface-area scaffold and π-conjugated platform; the metal–polyphenol network introduces abundant binding sites and enhances interfacial interactions; TiO_2_ increases surface polarity and promotes adsorption heterogeneity; whilst PNIPAm enables dynamic regulation of adsorption and desorption. Consequently, its exceptionally high adsorption capacity stems from the synergistic effects of chemical interactions and structural advantages.

**Figure 11:**
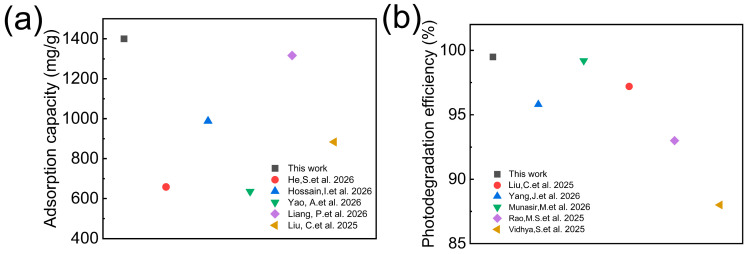
Comparison of TPAGA (1:1) with previously reported literature: (**a**) comparison chart of adsorption capacity; (**b**) comparison of degradation efficiency [40,41,42,43,44,45,46,47,48].

The simultaneous retention of high adsorption stability and photocatalytic efficiency was attributed to the synergistic structural features of TPAGA. As discussed in Figure 4, Figure 5 and Figure 6, the anisotropic graphene–aerogel framework provided mechanical integrity and continuous transport pathways, while the metal–polyphenol network effectively anchored TiO_2_ nanoparticles and PNIPAm chains, suppressing nanoparticle detachment and aggregation during repeated adsorption–desorption and photocatalytic cycles. The preserved interfacial contact and charge transfer efficiency contributed to the sustained photocatalytic activity. Overall, the results presented in Figure 10 demonstrated that TPAGA exhibited outstanding temperature-responsive adsorption–desorption stability and long-term photocatalytic durability, highlighting its strong potential for practical wastewater treatment applications involving repeated adsorption, recovery, and light-driven degradation processes. As shown in Figure 11, the performance of our composite material in terms of adsorption capacity and photodegradation is compared with values reported in previous studies [40,41,42,43,44,45,46,47,48]. Compared with other materials, the TPAGA_(1:1)_ composite we report exhibits superior performance in both adsorption and photocatalytic degradation.

It should be noted that the photocatalytic experiments in this work were carried out under controlled laboratory conditions using MB as a model pollutant, in order to elucidate the influence of pore structure, interfacial anchoring, and TiO_2_ loading on the catalytic behavior of the TPAGA system. In practical industrial wastewater, the photocatalytic process may be affected by additional factors such as coexisting ions, dissolved organic matter, turbidity, fluctuating pH, and variable light intensity, which may reduce the apparent degradation efficiency. Nevertheless, the excellent structural stability, strong interfacial immobilization of TiO_2_, and outstanding cycling durability demonstrated in this work suggest that the present system provides a robust platform for further studies under more realistic wastewater conditions. Such investigations will be carried out in future work to assess practical applicability more comprehensively.

## 4. Conclusions

This study rationally constructed a multifunctional anisotropic graphene–aerogel system integrating a metal–polyphenol network, TiO_2_ nanoparticles, and thermoresponsive PNIPAm chains through ice-template-directed freezing, controlled reduction, and in situ polymerization techniques. By regulating freezing temperatures and reduction conditions, the pore size distribution and specific surface area of the anisotropic graphene aerogel were effectively customized, yielding a highly accessible porous framework with optimized mass transport pathways and abundant interfacial sites. Systematic structural characterization confirmed the successful formation of a hierarchical anisotropic structure, stable metal-phenol coordination, and uniform incorporation of TiO_2_ and PNIPAm within the aerogel skeleton. Experiments revealed that photocatalytic degradation of methylene blue significantly depends on TiO_2_ loading, with a TiO_2_:GO = 1:1 ratio identified as optimal. At this ratio, visible-light irradiation for 60 min achieved over 99.5% photodegradation efficiency, simultaneously enabling efficient interfacial electron transfer and suppressing charge recombination. Excessive TiO_2_ loading induces nanoparticle aggregation, reducing photocatalytic efficiency. The composite material also exhibits superior adsorption–desorption performance, with an adsorption capacity of 28,000 mg/g below the LCST. Heating above the LCST enables desorption of 90.2% of the wastewater. Repeated photocatalytic cycling tests demonstrate exceptional long-term durability: degradation efficiency remains above 97% after 100 cycles. Furthermore, temperature-responsive adsorption–desorption experiments confirm the material’s high reversible stability over 100 cycles, with adsorption capacity retention exceeding 98%. The normalized adsorption capacity (Q/Q_0_) exhibits negligible decay during repeated thermal cycling, demonstrating the robust recovery capability conferred by the thermoresponsive polymer component. The synergistic interaction of anisotropic porous structure, stable interfacial anchoring, and thermoresponsive functionality enables an integrated wastewater treatment strategy encompassing adsorption, recovery, and light-driven degradation. This study provides an effective structural design approach for developing advanced aerogel platforms with tunable porosity, high stability, and multifunctionality, laying the foundation for sustainable wastewater management.

## Figures and Tables

**Table 1:** Concentrations and ratios of the various components of TPAGA_(x:y)_.

Sample	GO (mg/mL)	TiO_2_ (mg/mL)	MPN (%)	PNIPAm (%)
TPAGA_(4:1)_	4	16	10	10
TPAGA_(2:1)_	4	8	10	10
TPAGA_(1:1)_	4	4	10	10
TPAGA_(1:2)_	4	2	10	10

**Table 2:** Atomic percentage of different elements.

Sample	C (%)	O (%)	Ti (%)	C/O
GO	33.30	66.70	0	0.51
AGA	83.11	16.89	0	4.92
TPAGA_(1:1)_	43.94	46.18	9.88	0.95

## Data Availability

The original contributions presented in this study are included in the article. Further inquiries can be directed to the corresponding author.

## References

[1] Tara N., Siddiqui S.I., Rathi G., Chaudhry S.A., Inamuddin, Asiri A.M. (2020). Nano-engineered adsorbent for the removal of dyes from water: A review. Curr. Anal. Chem..

[2] Deniz F., Ersanli E.T. (2022). A novel biowaste-based biosorbent material for effective purification of methylene blue from water environment. Int. J. Phytoremediat..

[3] Emmanuel S.S., Adesibikan A. (2021). A Bio-fabricated green silver nano-architecture for degradation of methylene blue water contaminant: A mini-review. Water Environ. Res..

[4] Ayalew A.A., Aragaw T.A. (2020). Utilization of treated coffee husk as low-cost bio-sorbent for adsorption of methylene blue. Adsorpt. Sci. Technol..

[5] Razali M.H., Fauzi M.A.F.M., Azam B.M., Yusoff M. (2022). G-c3n4/tio2 nanocomposite photocatalyst for methylene blue photodegradation under visible light. Appl. Nanosci..

[6] Li S., Huang L., Zhang H., Huang Z., Jia Q., Zhang S. (2021). Adsorption mechanism of methylene blue on oxygen-containing functional groups modified graphitic carbon spheres: Experiment and dft study. Appl. Surf. Sci..

[7] Wang S., Huang H., Liu J., Deng Y. (2022). Micro-meso porous biocarbons derived from a typical biopolymer with superior adsorption capacity for methylene blue dye and high-performance supercapacitors. J. Electroanal. Chem..

[8] Srinivasan R. (2023). A sustainable and cyclic metal organic framework-driven fenton process for efficient removal of methylene blue. Inorg. Chem. Commun..

[9] Hamri N., Imessaoudene A., Hadadi A., Cheikh S., Boukerroui A., Bollinger J.-C., Amrane A., Tahraoui H., Tran H.N., Ezzat A.O. (2024). Enhanced adsorption capacity of methylene blue dye onto kaolin through acid treatment: Batch adsorption and machine learning studies. Water.

[10] Mahmoodi N.M., Soroush S., Bagherzadeh S.B., Mahmoodi B., Hayati B., Sorkheh M. (2025). Ternary mil-101 (fe)/tio2/go composites: Synthesis, characterization, and photocatalytic performance. Inorg. Chem. Commun..

[11] Verma N., Kumar V., Kesari K.K. (2022). Microbial and lignocellulosic biomass based dye decolourization. Biomass Convers. Biorefin..

[12] Ouzani A., Maachou H., Touzout N., Moussa H., Zouambia Y., Ainas M., Mihoub A., Prisa D., Černý J., Dewir Y.H. (2025). Hydroxyapatite pretreatment alleviates methylene blue phytotoxicity in wheat (*Triticum aestivum* L.) seedlings. Int. J. Phytoremediat..

[13] Li C., Chen D., Ding J., Shi Z. (2020). A novel hetero-exopolysaccharide for the adsorption of methylene blue from aqueous solutions: Isotherm, kinetic, and mechanism studies. J. Clean. Prod..

[14] Ren H., Long G., Chen Y., Gao Y., Mao H., Gong X., Xue D., Hu G. (2024). Enhanced methylene blue degradation by graphene oxide-encapsulated nano zero-valent iron composites: Optimization, mechanistic insights, and environmental implications. Mater. Sci. Semicond. Process..

[15] Chavan R.R., More V.R., Pawar N.V., Dawkar V.V., Jadhav J.P., Patil R.B., Chougale A.D. (2025). Catalytic and kinetic studies of cufe2o4 as a superior heterogeneous nanocatalyst for dye degradation and cr (vi) reduction. Clean Technol. Environ. Policy.

[16] Weng R., Tian F., Yu Z., Ma J., Lv Y., Xi B. (2021). Efficient mineralization of tbbpa via an integrated photocatalytic reduction/oxidation process mediated by mos2/snin4s8 photocatalyst. Chemosphere.

[17] Samuel P.J., Thinley T., Vinod D., Anusha H.S., Hemanth Vikram P.R., Gurupadayya B.M., Anilkumar K.M., Selvaraj M., Assiri M.A., Shivaraju H.P. (2024). Novel ti/rgo@ zncr ldh derived mmo for photocatalytic conversion of atmospheric elements and organic pollutants into valuable resources. J. Environ. Chem. Eng..

[18] Zeghioud H., Hamdaoui O., Amrane A. (2025). Advanced nanomaterials for green hydrogen production: A review on tio2 nanotube-based composites, technologies, challenges, and vosviewer bibliometric analysis. Int. J. Hydrogen Energy.

[19] Chauke N.M., Ngqalakwezi A., Raphulu M. (2025). Transformative advancements in visible-light-activated titanium dioxide for industrial wastewater remediation. Int. J. Environ. Sci. Technol..

[20] Haynes V.N., Ward J.E., Russell B.J., Agrios A.G. (2017). Photocatalytic effects of titanium dioxide nanoparticles on aquatic organisms—Current knowledge and suggestions for future research. Aquat. Toxicol..

[21] Fujishima A., Honda K. (1972). Electrochemical photolysis of water at a semiconductor electrode. Nature.

[22] Amorim S.M., Steffen G., Junior J.M.d.S., Brusamarello C.Z., Romio A.P., Domenico M.D. (2021). Synthesis, characterization, and application of polypyrrole/tio2 composites in photocatalytic processes: A review. Polym. Polym. Compos..

[23] Sakar M., Prakash R.M., Do T.-O. (2019). Insights into the tio2-based photocatalytic systems and their mechanisms. Catalysts.

[24] Shaik B.B., Katari N.K., Raghupathi J.K., Jonnalagadda S.B., Rana S. (2024). Titanium dioxide/graphene-based nanocomposites as photocatalyst for environmental applications: A review. ChemistrySelect.

[25] Shahid M.U., Muhsan A.S., Saheed M.S.M., Azeem B., Zaine S.N.A., Ahmad W., Shahid M.Z., Rahman M.H. (2025). Graphene-modified photoelectrodes for efficient dye-sensitized solar cells: A review. Energy Fuels.

[26] Giovannetti R., Rommozzi E., Zannotti M., D’Amato C.A. (2017). Recent advances in graphene based tio2 nanocomposites (gtio2ns) for photocatalytic degradation of synthetic dyes. Catalysts.

[27] de Abajo F.J.G. (2014). Graphene plasmonics: Challenges and opportunities. ACS Photonics.

[28] Huang C.-Y., Lin Y.-C., Chung J.H.Y., Chiu H.-Y., Yeh N.-L., Chang S.-J., Chan C.-H., Shih C.-C., Chen G.-Y. (2023). Enhancing cementitious composites with functionalized graphene oxide-based materials: Surface chemistry and mechanisms. Int. J. Mol. Sci..

[29] Ramachandran T., Roy N., Hegazy H., Yahia I., Kumar Y.A., Moniruzzaman M., Joo S.W. (2025). From graphene aerogels to efficient energy storage: Current developments and future prospects. J. Alloys Compd..

[30] Park J., Yan M. (2016). Three-dimensional graphene-tio2 hybrid nanomaterial for high efficient photocatalysis. Nanotechnol. Rev..

[31] Huang H., Shi H., Das P., Qin J., Li Y., Wang X., Su F., Wen P., Li S., Lu P. (2020). The chemistry and promising applications of graphene and porous graphene materials. Adv. Funct. Mater..

[32] Wu Y., An C., Guo Y., Zong Y., Jiang N., Zheng Q., Yu Z.-Z. (2024). Highly aligned graphene aerogels for multifunctional composites. Nano-Micro Lett..

[33] Shahbazi M.-A., Ghalkhani M., Maleki H. (2020). Directional freeze-casting: A bioinspired method to assemble multifunctional aligned porous structures for advanced applications. Adv. Eng. Mater..

[34] Garcia-Bordejé E., Benito A.M., Maser W.K. (2021). Graphene aerogels via hydrothermal gelation of graphene oxide colloids: Fine-tuning of its porous and chemical properties and catalytic applications. Adv. Colloid Interface Sci..

[35] Li Y., Luo J., Xie G., Zhu D., Zhao C., Zhang X., Liu M., Wu Y., Guo Y., Yu W. (2024). Recent progress on regulating the lcst of pnipam-based thermochromic materials. ACS Appl. Polym. Mater..

[36] Marcano D.C., Kosynkin D.V., Berlin J.M., Sinitskii A., Sun Z., Slesarev A., Alemany L.B., Lu W., Tour J.M. (2010). Improved synthesis of graphene oxide. ACS Nano.

[37] Chen J., Yao B., Li C., Shi G. (2013). An improved hummers method for eco-friendly synthesis of graphene oxide. Carbon.

[38] Kuilla T., Bhadra S., Yao D., Kim N.H., Bose S., Lee J.H. (2010). Recent advances in graphene based polymer composites. Prog. Polym. Sci..

[39] Stetsyshyn Y., Ohar H., Budkowski A., Lazzara G. (2025). Molecular design and role of the dynamic hydrogen bonds and hydrophobic interactions in temperature-switchable polymers: From understanding to applications. Polymers.

[40] He S., Tang J., Sheng H., Zhu J., Cao C., Huang Y. (2026). Multi-active-site uio-66-no2-based carboxymethyl cellulose-sodium alginate composite aerogel: Efficient adsorption of methylene blue and methyl orange. Int. J. Biol. Macromol..

[41] Hossain I., Nie K., Iliut M., Vijayaraghavan A. (2026). Core-shell graphene oxide and carboxymethyl cellulose vortex ring aerogel particles for the adsorptive removal of contrasting azo dyes from water. J. Mol. Liq..

[42] Yao A., Fu X., Xu Q., Liu C., Mo Y., Xue Q., Shang J., Lan J., Lin S. (2026). Magnetic co2-responsive aerogels with ag/fe mof-derived carbon for adsorption and photo-fenton-like degradation of organic pollutants. Desalination.

[43] Liang P., Ain Q.U., Dai L., Huang K., Huang K., Tong Z. (2026). Eco-engineered sodium alginate-bentonite-fe-ata aerogels with tunable crosslinking for high-efficiency cationic dye adsorption. Int. J. Biol. Macromol..

[44] Liu C., Yao A., Li W., Xu Q., Yang L., Ge Y., Lan J., Lin S., Qiu J. (2025). Design of go@ tio2 and pda@ cnc decorated gelatin aerogel for efficient adsorption and photocatalytic degradation of organic pollutants. J. Water Process Eng..

[45] Yang J., Jia W., Wu L., Huang L., Xiao T., Wang B., Sheng X., Sun Y., Fatehi P., Shi H. (2026). Compressible lignin-enhanced holocellulose aerogel for adsorption applications. Carbohydr. Polym..

[46] Munasir M., Okto S.H.S., Faaizatunnisa N., Ariesta M.N., Suaebah E., Anggaryani M., Taufiq A., Diantoro M., Hardianto Y.P. (2026). Phytochemical-assisted synthesis of tio2/graphene oxide nanocomposite using syzygium cumini for enhanced photocatalytic degradation of organic pollutants in aqueous systems. J. Environ. Sci..

[47] Rao M.S., Chaudhary Y., Jaihindh D.P., Lin Y.-F., Rakesh B., Sankaran K.J. (2025). Zno-graphene nanohybrids for photocatalytic degradation of methylene blue dye. Diam. Relat. Mater..

[48] Vidhya S., Subramanian Y., Gajendiran J., Raj S.G., Bharath Sabarish V.C., Durairajan A., Le M.T., Mamudu U., Kumar G.R., Kumar J.K. (2025). Methylene blue dye degradation characteristics of bifeo3-graphene-linbo3 ternary nanocomposites. Sustain. Mater. Technol..

